# Ethical and academic dilemmas in authorship of clinical research publications: a medical oncologist’s perspective

**DOI:** 10.1007/s12032-025-02617-4

**Published:** 2025-02-11

**Authors:** Ozgur Tanriverdi

**Affiliations:** Department of Medical Oncology, Mugla Sıtkı Koçman University Faculty of Medicine, Kötekli Mh. Marmaris Yolu Bulvarı No: 55 Menteşe, 48000 Muğla, Türkiye

**Keywords:** Clinical trials, Authorship, Ethics, Perspective

## Abstract

Authorship in clinical research carries significant academic, financial, and social implications. However, determining rightful authorship often introduces ethical and professional dilemmas, particularly in large, multidisciplinary studies, such as those common in oncology. This article explores the ethical complexities surrounding authorship in clinical research from the viewpoint of a medical oncologist. It addresses issues, such as academic jealousy, the inclusion of industry-affiliated researchers, honorary authorship, and the role of patient recruitment in authorship qualification. By examining current guidelines, ethical considerations, and practical cases, this paper aims to offer insights into fostering fair and transparent authorship practices in the field of clinical oncology.

## Introduction

In oncology, clinical research often involves large, diverse teams spanning clinicians, statisticians, laboratory scientists, and industry partners. The process of determining authorship in this complex setting raises significant ethical and academic concerns. While guidelines such as those from the International Committee of Medical Journal Editors (ICMJE) provide clear standards, applying these consistently is challenging [1]. Authorship disputes can lead to strained relationships, loss of trust, and even allegations of misconduct [3–6].

Authorship disputes in clinical research are a recurring issue across scientific fields, yet they are particularly prevalent in oncology, where clinical trials often involve multiple centers, stakeholders, and significant financial interests [7, 8]. As a medical oncologist, the pressure to publish, coupled with the collaborative nature of the field, creates a landscape rife with ethical dilemmas. Beyond individual ambition, authorship can shape careers, funding opportunities, and professional recognition.

Despite the clear guidelines from organizations such as the ICMJE, disputes over authorship persist [1–4]. This article explores the various facets of authorship dilemmas, including the role of academic rivalry, undue inclusion or exclusion of authors, and the influence of pharmaceutical companies. It highlights the need for integrity and transparency to ensure the credibility of clinical oncology research.

### The framework of ethical authorship and ıts role in clinical research publications

ICMJE has established four primary criteria for authorship to ensure transparency, accountability, and fairness in academic publications, particularly in clinical research [3–5]. Each of these criteria addresses key ethical concerns that commonly arise in collaborative studies, such as oncology trials, where diverse contributions must be carefully evaluated.

#### Significant contribution to the conception or design of the work, data collection, or analysis

The first criterion emphasizes the importance of intellectual and practical involvement in the early stages of research. This includes designing the study, formulating hypotheses, and collecting and analyzing data. In large-scale clinical trials, contributions can vary widely, raising questions about the inclusion of individuals who primarily recruit patients but do not engage in data interpretation or experimental design. Patient recruitment is often conducted by clinicians who may have limited input in manuscript preparation. Despite their crucial role in trial success, these contributors sometimes fall short of fulfilling all authorship requirements. Literature suggests that while patient recruitment alone should not warrant authorship, significant involvement in protocol development or critical analysis should. Additionally, multidisciplinary trials increasingly require correlative science, such as biomarker analysis and genetic profiling. Researchers contributing to these aspects are vital to the study’s integrity, but their inclusion in the author list may be debated if their work is restricted to sample collection [9].

In correlative science, tissue biopsies or genetic data play a critical role in understanding treatment responses. While laboratory scientists may not directly interact with patients, their contributions to the study design and data analysis justify their inclusion as authors under ICMJE guidelines [10].

#### Drafting or revising the manuscript for ıntellectual content

Writing or critically revising a manuscript involves distilling data into coherent findings, interpreting results, and contributing novel insights. This criterion ensures that listed authors actively shape the final product rather than passively contribute raw data or samples. Research by the CHARM team at Kaiser Permanente emphasized the importance of clearly defining manuscript roles at the outset of a study. Their authorship guidelines recommend regular reviews of contributions to ensure all team members involved in drafting or revising the manuscript are appropriately credited. The study further highlights the value of assigning sections to various contributors, promoting equity across multidisciplinary teams [11]. Similarly, Leung underscores that failure to involve all contributors in manuscript drafting can lead to ghost authorship, an unethical practice that undermines research integrity [12].

Large consortia now often require contributors to submit written summaries of their involvement during manuscript development. This approach promotes fairness and ensures that revisions and intellectual contributions are documented and recognized [13].

#### Approval of the final manuscript before publication

Final manuscript approval ensures that all authors agree on the presented data, interpretations, and conclusions. This criterion holds each author accountable for the integrity and accuracy of the publication, reducing the risk of errors or misinterpretations slipping through the review process. A systematic review by Schroter et al. suggests that requiring unanimous approval fosters collective responsibility, strengthening the overall integrity of clinical publications. However, it also highlights that in some instances, senior authors may approve manuscripts without adequately consulting junior researchers, leading to ethical discrepancies [14]. Another study by Tsang notes that fragmented contributions, especially in multicenter trials, complicate final approval [15]. To mitigate this, research teams often implement shared online platforms that allow all authors to review and suggest changes in real time. In multicenter oncology trials, it is common practice for the principal investigator to coordinate the final manuscript review process, ensuring that all listed authors have access to the document and can propose revisions before submission [13–16].

#### Accountability for the ıntegrity of the work and the accuracy of reported findings

Accountability is the cornerstone of ethical authorship. This criterion ensures that all authors are responsible for the content of the publication, addressing any questions or concerns about the study’s validity. Authors must also be willing to engage in post-publication corrections or retractions if necessary. The issue of accountability is particularly relevant in oncology trials, where erroneous findings can have direct clinical implications. A report by Petersen et al. on large-scale cancer trials stresses that authorship should not merely reflect contribution but also a willingness to defend the data if challenged [17]. The report highlights that in high-stakes research, junior researchers or data analysts may hesitate to question senior authors, potentially allowing flawed data to go uncorrected. Further, accountability should extend beyond the published paper, with authors committing to ongoing transparency regarding trial data, adverse events, and long-term follow-up results. A notable example involves a large-scale Phase III oncology trial in which discrepancies in survival data emerged post-publication. Authors were required to collectively investigate and issue a correction, reinforcing the importance of shared responsibility in clinical research [18].

The ICMJE’s four-criterion framework provides a robust ethical foundation for authorship in clinical oncology research. However, practical implementation can be challenging, particularly in multidisciplinary and multicenter studies. Addressing these dilemmas requires transparent communication, explicit authorship agreements, and ongoing education on publication ethics. By adhering to these principles, research teams can uphold scientific integrity and ensure fair recognition for all contributors. This framework aims to balance fairness with responsibility, preventing unjustified authorship while promoting accountability [1, 3, 13–16]. Despite these standards, practical implementation is often complex, particularly in large oncology trials involving numerous investigators [14].

### Possible situations creating dilemmas

#### The role of patient recruitment

A contentious issue in oncology research is whether patient recruitment alone qualifies a clinician for authorship. In multicenter trials, some investigators contribute primarily by enrolling patients but play little role in data analysis or manuscript preparation. While recruitment is crucial, the ICMJE guidelines emphasize intellectual contributions over logistical roles. However, excluding these clinicians risk alienating key collaborators, potentially jeopardizing trial recruitment. A balanced approach involves recognizing such contributions in the acknowledgments section while reserving authorship for those involved in the manuscript’s intellectual development [19].

#### Honorary and ghost authorship

Honorary authorship – where individuals are listed without significant contributions – remains widespread, often driven by hierarchical pressures or institutional prestige. Conversely, ghost authorship occurs when substantial contributors are omitted, typically to obscure industry involvement or minimize perceived conflicts of interest. Both practices compromise research integrity. Honorary authorship can inflate the perceived contribution of senior researchers, while ghost authorship conceals potential biases, undermining the credibility of the findings [20].

#### The influence of ındustry and external stakeholders

Pharmaceutical and biotechnology companies frequently fund oncology trials, raising questions about the independence of researchers and the integrity of authorship. Contracts may stipulate the inclusion of industry-affiliated authors or limit the researchers’ ability to publish negative results. Ethical frameworks advocate for complete transparency regarding industry involvement. Researchers must retain the right to publish all findings, regardless of commercial interests. Journals increasingly require disclosure of all contributors, including those involved in data analysis, to mitigate the risk of hidden conflicts [21].

#### Authorship and multidisciplinary teams

Oncology research often involves collaboration between clinicians, biostatisticians, laboratory scientists, and data managers. Disputes frequently arise over the relative contributions of these diverse roles. While clinicians may drive patient care and data collection, basic scientists and statisticians provide essential analytical insights, warranting equal recognition. Instituting clear authorship agreements at the project’s inception can prevent disputes. Regular review and transparent communication about evolving contributions ensure that all collaborators feel valued and recognized [22–24].

### Possible academic consequences of dilemmas

#### Academic jealousy and competition

Academic environments, particularly in competitive fields like oncology, can foster jealousy and rivalry. Researchers may downplay the contributions of colleagues to minimize their visibility or delay publications to prevent others from gaining recognition. This competitive atmosphere can undermine collaboration, resulting in fractured research teams and questionable ethical decisions. Case studies reveal instances where junior researchers or clinicians are excluded from authorship despite significant contributions to patient recruitment or data collection. Such exclusion can lead to resentment, disengagement, and even misconduct [24, 25].

#### Academic anxiety and publish-or-perish culture

The pressure to publish frequently leads researchers to pursue multiple authorships, sometimes at the expense of quality. This “publish-or-perish” culture exacerbates unethical practices, fostering an environment where individuals prioritize publication volume over scientific rigor. Junior oncologists, particularly those pursuing tenure, may feel compelled to accept or demand authorship on papers where their contributions are minimal. Addressing this requires institutional change, emphasizing the quality and impact of research rather than sheer publication quantity [25, 26].

#### Recommendations

Ethics committees and institutional review boards play a crucial role in mediating authorship disputes. Establishing clear policies and encouraging open dialog can help resolve conflicts early, preserving collaborative relationships. Training programs focusing on publication ethics can further cultivate a culture of integrity, ensuring that all researchers understand and adhere to authorship guidelines (Table 1).

**Table 1:** Ethical dilemmas in authorship of clinical research publications and proposed solutions

Type of dilemma	Underlying cause	Potential consequences	Proposed solutions
Patient recruitment (Inclusion in authorship)	Contribution limited to patient enrollment	Disengagement, reduced collaboration	Acknowledge contributions in the acknowledgment section; provide opportunities for manuscript involvement
Honorary authorship	Seniority pressure, institutional hierarchy	Erodes trust, inflates credentials	Implement transparent authorship contribution forms; enforce ICMJE criteria strictly
Ghost authorship	Industry involvement, desire to minimize conflicts	Undermines credibility, hides potential biases	Mandate full disclosure of all contributors; encourage journals to require transparency
Multidisciplinary research teams	Unequal contributions from various disciplines	Tensions between clinical and non-clinical staff	Establish authorship agreements at project inception; conduct periodic reviews of contributions
Industry influence	Sponsorship-driven author list inclusions	Compromised objectivity, publication bias	Maintain researcher autonomy over publications; require full disclosure of financial ties
Academic rivalry/Jealously	Competitive atmosphere in academic settings	Exclusion of contributors, delayed publications	Foster a culture of collaboration and openness; encourage ethics training
Publish-or perish culture	Pressure to publish frequently	Quantity over quality, diluted research rigor	Shift focus to research quality over volume; adjust institutional performance metrics
Fragmented contributions	Large multicenter trials with scattered inputs	Uneven authorship recognition	Use collaborative platforms for shared manuscript development; require section contributions from all authors
Lack of final approval participation	Senior authors approving without junior input	Junior author exclusion, errors overlooked	Require documented final approval from all authors; use online platforms for transparent manuscript reviews
Discrepancies in accountability	Hesitancy of junior authors to challenge seniors	Flawed data, ethical breaches	Promote shared responsibility through collective sign-off; foster mentorship and open dialog

## Conclusion

Authorship dilemmas in clinical oncology reflect broader challenges within academic medicine. By adhering to ethical frameworks, fostering transparent communication, and prioritizing collaboration over competition, researchers can mitigate conflicts and ensure fair recognition for all contributors. Upholding authorship integrity is not only a professional obligation but also a cornerstone of advancing medical science and patient care.

In clinical research, the design and methodological framework of a study are typically established during the initial phases, often by a core group of investigators, statisticians, and methodologists. By the time patient recruitment begins, the structural and scientific aspects of the trial are largely set, limiting opportunities for clinicians involved in patient enrollment to contribute meaningfully to the study’s design or data interpretation. This discrepancy can create ethical dilemmas regarding authorship, as patient recruiters play a critical logistical role but may not fulfill the intellectual contributions required by authorship criteria, such as those outlined by the ICMJE. Literature highlights that while patient recruitment is essential to the success of clinical trials, it alone does not justify authorship unless accompanied by substantial involvement in protocol development, data analysis, or manuscript drafting. This gap can lead to tensions within research teams, risking disengagement or disputes over recognition. Acknowledging such contributions in sections other than the author list, such as the acknowledgments, is often recommended to balance fairness with academic integrity.

## Data Availability

No datasets were generated or analyzed during the current study.
